# Post-Transcriptional and Epigenetic Regulation of Antigen Processing Machinery (APM) Components and HLA-I in Cervical Cancers from Uighur Women

**DOI:** 10.1371/journal.pone.0044952

**Published:** 2012-09-14

**Authors:** Ayshamgul Hasim, Mangnishahan Abudula, Reshalaiti Aimiduo, Jun-Qi Ma, Zhen Jiao, Gulzareye Akula, Ting Wang, Abulizi Abudula

**Affiliations:** 1 Department of Pathology, College of Basic Medicine, Medical University of Xinjiang, Urumqi, People’s Republic of China; 2 . Oncology Center of the First Affiliated Hospital, Medical University of Xinjiang, Urumqi, People’s Republic of China; 3 Department of Gynecology in the First Affiliated Hospital, Medical University of Xinjiang, Urumqi, People’s Republic of China; 4 Department of Gynecology in the Hospital of Xinjiang Uighur Autonomous Region, Urumqi, People’s Republic of China; 5 Key Laboratories of Molecular Biology and Endemic Diseases of Xinjiang Uighur Autonomous Region, Medical University of Xinjiang, Urumqi, People’s Republic of China; Karolinska Institutet, Sweden

## Abstract

Normal function of human leukocyte antigen class I (HLA-I) and antigen processing machinery (APM) proteins is required for T cell-mediated anti-tumor or antiviral immunity, whereas the tumor survival indicates a failure of the host in immune surveillance associated with the dysfunction in antigen presentation, mainly due to the deregulation in HLA-I and APM expression or function. The posttranscriptional regulation of HLA-I and APM expression may associate with epigenetic modifications in cancer development which was not described so far. Here we showed that the development of cervical intraepithelial neoplasia (CIN) and cervical squamous cell carcinoma (CSCC) in Uighur women was accompanied with the partial or total loss of protein expression of HLA-I, ß2-m and APM components, including the transporter associated with antigen processing (TAP1/2), low molecular mass protein (LMP2, LMP7), endoplasmic reticulum aminopeptidase 1(ERAP1), chaperone molecules include calreticulin (CLR), calnexin (CNX) and ERp57, and this was proved again by analysis of transcription of the same genes in addition to three genes HLA-A, B and C coding for HLA-I. By bisulfite sequencing approach, we identified target CpG islands methylated at the gene promoter region of TAP1, TAP2, LMP7, tapasin and ERp57 in cervical carcinoma cells. Further analysis of CpG site specific methylation of these genes in cases of CSCC and CIN demonstrated an inverse correlation of altered CpG island methylation of TAP1, LMP7, and ERp57 with changes in protein expression. Moreover, promoter methylation of these genes was significantly higher in cases positive for human papillomavirus 16 (HPV 16) than negative ones. Our results suggested that epigenetic modifications are responsible for the aberrant expression of certain HLA-I and APM genes, and may help to understand unrevealed mechanisms of tumor escape from immune surveillance in cervical carcinogenesis.

## Introduction

The formation and survival of a tumor cell is a sign of successful immune escape and a failure in host immune surveillance and elimination. Normal function of human leukocyte antigen class I (HLA-I) and antigen processing machinery (APM) is a prerequisite for T cell-mediated innate and adaptive immune responses against viral infection or cellular carcinogenesis [1]–[3]. HLA-I is a central player and works together with APM members in assembling, loading, and presenting endogenous antigenic peptide as well as regulating cellular and humoral immunity [4]–[5]. Thus, elimination of tumor cells by the immune system greatly depends on the expression of APM and HLA-I.

In recent years, progress has been made in understanding how peptides presented by HLA-I molecules. In particular, it is known which proteases are involved and how intracellular pathways influence antigen presentations in professional antigen presenting cells and in various types of malignant disease [6]–[8]. The APM of the cell is a complex system of interacting proteins, including proteasome subunits, low-molecular-mass proteins 2 and 7(LMP2 and LMP7), transporters associated with antigen processing 1 and 2 (TAP1 and TAP2), endoplasmic reticulum aminopeptidase 1 (ERAP1), and the ER chaperone proteins calnexin, calreticulin, and tapasin. Additionally, the thiol oxidoreductase ERp57 is responsible for presentation of HLA-I peptide complexes on the cell surface and is crucial for recognition of virally infected or malignantly transformed cells, maintenance of self-tolerance, and surveillance of newly arising tumors by the immune system [9]. Downregulation of HLA-I and APM components has been observed in many tumors and is closely associated with tumor immunoevasion, growth, and metastatic ability [6]–[8]. The molecular mechanisms underlying the relatively low level of transcription of HLA-I and APM components in cancer cells are largely unexplained. Epigenetic modifications of the human genome, including aberrant DNA methylation, represent tumor genetic events that are functionally equivalent to genetic changes. Downregulation of HLA-I expression caused by hypermethylation of HLA-I genes has been documented in esophageal squamous cell carcinoma, colon carcinoma, and melanoma cell lines, and this could be reversed upon treatment with DNA methyltransferase inhibitors [10]–[11].

Human papillomavirus (HPV) infection plays a central role in the pathogenesis of cervical intraepithelial neoplasia (CIN) and cervical cancer with HPV infection considered to be a necessary, but not always sufficient, cause [12]. Development of human cervical cancer without involvement of a specific HPV is exceptional, and it is widely accepted that in addition to HPV infection, other cofactors, including endogenous hormones, genetic factors such as HLA-I, and other factors related to the host’s immune response, may have important roles in the development of cervical lesions [13].

The cervical cancer in Uighur has been occurred with high morbidity and mortality women in Xinjiang region and considered as a high incident disease of the region in China [14]. HPV infection is believed to be the main cause of cervical cancer development, and the infection of the virus, especially the high-risk HPVs is detectable in Uighur women with cervical cancer at a rate comparable with other populations in and outside of China [15]. Our previous studies have shown that the tendency in the loss rate of HLA-I protein expression was comparable for both women from Uighur and Han ethnic groups in cervical cancer development, but the total loss rate of HLA-I in specimens from cervical squamous cell carcinoma (CSCC) and its precursor lesions (CIN II or III) was higher in Uighur women (27% and 53%, respectively) than in Han women (18% and 37%, respectively) [16]. It seems that ethnic differences may have, to some extent, influence on tumor development due to the different genetic background, living habits or geographical environment. Therefore, considering that aberrant surface expression of HLA-I in cervical cancer associated with high-risk HPV16 infection frequently caused by the absence of APM [17]–[18], one would ask whether epigenetic modifications would modulate the surface expression of HLA-I directly or indirectly by silencing or inactivating of APM component genes and lead to the development of cervical cancer in Uighur women.

In this study, we focused on 10 candidate genes belong to the HLA-I and APM family, analyzed the association of cancer development with altered gene expression at different levels, including the gene promoter methylation, transcription, and protein expression. These data provide insight into unknown mechanisms of cervical carcinogenesis in relation to the regulation HLA-I and APM expression and may provide with molecular marker profile for early diagnosis based on the detection of epigenetic modifications and expression of these genes. In addition, this study allows further understanding of the impact of HLA-I mediated antigen presentation in antiviral or anti-tumor immunity or abnormalities caused by, or leading to, cervical carcinogenesis.

## Materials and Methods

### Clinical Characteristics and Tissue Samples

Fresh or formalin-fixed and paraffin-embedded cervical tissue specimens were collected from Uighur women with cervical squamous cell carcinoma (CSCC) and cervical intraepithelial neoplasia (CIN), or without cervical diseases, but treated by hysterectomy. All cervical cancer patients referred between June 2009 and March 2010 because of cervical cancer were asked to participate in our study during their initial visit to the Department of Gynecology of the First Affiliated Hospital in Medical University of Xinjiang. Informed consent was obtained from all patients and controls participating in this study. The study was approved by the ethics committee of our hospital.

Gynecological examination was performed in all cervical cancer patients for staging in accordance with the International Federation of Gynecology and Obstetrics (FIGO) criteria. 152 cases of formalin-fixed, paraffin-embedded (FFPE) tissue specimens were obtained from the tissue sample archive of the pathological department after examination of archival slides by experienced pathologists. The FFPE specimens (or fresh frozen cervical tissues described below) were originally collected during the initial visit to the outpatient department or at gynecologic examination or after operative treatment under general anesthesia. Of patients with CSCC enrolled in this study, were 29 FIGO stage IIB, 25 FIGO stage IIIB, 9 FIGO stage IVB. Among them, 28 cases were pathologically characterized as well-differentiated, 19 moderately differentiated and 16 poorly differentiated tumors. Lymph node metastasis was documented for 31 tumor patients. A total of 64 patients with CIN were selected for this study, including 33 cases with CIN I-II and 31 with CIN III. The median age of the cervical cancer patients was 49.5 years (IQ range 29–67 years). Control cases (n = 25) were obtained from patients without a history of cervical lesions or any form of cancer and planned to undergo a hysterectomy for nonmalignant reasons during the same period. Indications for hysterectomy were fibroids, prolaps uteri or adenomyosis or mainly a combination of fibroids with prolaps uteri.

In addition, 55 frozen biopsies, including 20 cases of CIN, 20 CSCC and 15 control cases, were collected according to the criteria described above, for the detection of gene transcription by semi-quantitative reverse transcription-polymerase chain reaction (RT-PCR).

### Immunohistochemical Studies

Immunohistochemical (IHC) staining was performed with primary antibodies recognizing target proteins and IHC Kits containing biotin-labeled secondary antibodies. the mouse mAb HLA class I heavy chain(HC-10,1∶300; Santa Cruz Biotechnology, Santa Cruz, CA, USA)was recognized as ß2-m–free [19]. The rabbit polyclonal anti-b2-m-antibody was purchased from (1∶100; Shanghai Jingtian, Biotechnology, China), TAP1 and TAP2 antibody (1∶300, Santa Cruz Biotechnology, respectively), LMP2 and LMP7 antibody (1∶100; Abcam, Cambridge, MA, USA, respectively), ERAP1 antibody (1∶200; Abcam), ERp57 antibody (1∶300, Santa Cruz Biotechnology), calnexin and calreticulin antibody (1∶50, Shanghai Jingtian, Biotechnology, China, respectively), and tapasin antibody (1∶300, Santa Cruz Biotechnology). Briefly, 3-µm-thick sections were cut from the paraffin-embedded tissue blocks. After being dewaxed in xylene and rehydrated in alcohol and distilled water, antigen was retrieved by heating in the microwave oven for 15 min at 95°C in EDTA buffer (pH 8.0). After cooling and rinsing in distilled water, endogenous peroxidase activity was blocked by incubating sections for 15 min in 3% H_2_O_2_, followed by rinsing in 0.01 M PBS (pH 7.4) for 10 min. Samples were preincubated with a protein blocking solution for 10 min and the sections were incubated at 4°C overnight in a humid chamber with the indicated antibodies, Slides were washed three times in PBS and then incubated with a biotinylated secondary antibody (Zhong Shan Goldenbridge Biotechnology Co. Ltd., China) for 15 min at room temperature. The reaction products were visualized with diaminobenzidine (DAB Kit; Zhongshan Goldenbridge Biotechnology). PBS was used in place of the primary antibody as a negative control and slides were counterstained with hematoxylin, dehydrated, and evaluated under light microscope.

The percentage and intensity of positively stained tumor cells in each lesion was investigated by two pathologists who had no knowledge of the patients’ characteristics. A consensus number was reached for each tumor sample between the two investigators. Results were scored on a scale from 0 to 3 by the percentage and intensity of positive cells among tumor cells. The percentage of positive cells was scored as 0 for ≤10%, 1 for 11–25%, 2 for 26–50%, 3 for 51–75%, 4 for ≥76%. The intensity of staining is as follows: 0 indicates absence of staining, 1 indicates weak staining, 2 indicates moderate staining, and 3 indicates intense staining. The sum of both scores was used to identify three categories of expression: normal expression (total score 7–8), partial loss of expression (3–6), and total loss of expression (0–2). IHC staining demonstrated strong expression of HLA-I in stromal tissue and tumor-infiltrating inflammatory cells, thereby providing an internal positive control, as suggested by a previous study [20].

### RNA Extraction and Semi-quantitative RT-PCR

Total RNA was extracted from patients’ tissues using Trizol (Invitrogen, Carlsbad, CA, USA) as recommended by the manufacturer. The mRNA (1µg) was reverse transcribed into cDNA with RevertAid (MBI Fermentas, Burlington, Ontario, Canada) at 42°C for 60 min. The cDNA (20ng) was amplified by PCR in a 25µL mixture under following conditions: 95°C for 1 min, followed by 25 or 30 cycles of denaturing at 95°C for 30 sec, annealing at 60°C for 30 sec, and synthesis at 68°C for 1 min, and a final extension at 68°C for 10 min. PCR products were separated on a 4% agarose gel, visualized with GoldView I (Beijing Solarbio Co., Beijing, China) for ultraviolet detection and quantification by Gel Imaging System and professional software(GelDoc XR, Bio-Rad Laboratories, CA, USA). Each analysis was repeated at least twice to ensure reproduced mRNA, β-actin mRNA was used as an internal loading and normalization control. Forward and reverse primer pairs and their products are listed in Table S1.

### Cell Lines

The SiHa human carcinoma cells were obtained from the American Type Culture Collection (ATCC; Manassas, VA, USA). Cells were grown in RPMI 1640 media (Invitrogen) supplemented with 10% FCS, L-glutamine, and antibiotics (Sigma-Aldrich).

### DNA Extraction, Bisulfite Treatment, and Bisulfite-sequencing PCR (BSP)

DNA was extracted from SiHa cells using a DNA extraction kit (QIAGEN, Valencia, CA, USA), and genomic DNA (500 ng) was bisulfite-modified using the EZ Methylation Gold Kit (Zymo Research, Orange, CA, USA) according to the manufacturer’s instructions [21]. CpG island fragment specific primers were designed by scanning gene promoter regions using specialized Methyl Primer Express software(ABI company)based on genetic information obtained from the Genbank database. Bisulfite treated DNA from cervical cancer cells was PCR amplified. Primers used for subsequent amplifications are listed in Table S2. Complete bisulfite modification was confirmed by sequence analysis. BSP amplifications were performed in 50-µl reaction mixtures containing 2 µl bisulfite-modified genomic DNA, 2 µl dNTPs, 1.2 µl primers, 2 µl MgCl_2_, 20 nM ammonium sulfate,_._ 75 nM Tris-HCl (pH 8.3), and 3 U of Taq DNA polymerase. The touch-down PCR scheme was applied to amplify with the following cycling conditions: 95°C denaturation for 15 min, 95°C for 20 sec, annealing temperatures ranging from 62°C to 56°C for 1 min, extension at 72°C for 1 min for 45 cycles, and final incubation at 72°C for 7 min. Annealing temperatures were as follows: HLA-B: 60°C, TAP1∶62°C, TAP2∶56°C, LMP: 60°C, LMP2∶58°C, tapasin: 58°C, ERAP1∶62°C, and ERP57∶56°C. PCR was followed by cloning into vectors and sequencing to identify CpG sites related to gene promoter methylation.

### Genomic DNA Isolation and Quantitative DNA Methylation Analysis

Genomic DNA was extracted using a QIAamp DNA Mini Kit (QIAGEN, Valencia, CA). For quantitative detection of methylated DNA, MassARRAY (Sequenom, San Diego, CA, USA) was used to analyze the cervical tissue DNA for CpG content. Target gene specific primer pairs were used to compare methylation levels of target fragments and CpG sites among different samples according to the manufacturer’s instructions and as described previously [22]–[23]. The analyzed regions and CpG sites of candidate gene promoters are shown in Table S3. The primers used were designed according to Sequenom Standard EpiPanel (Sequenom, November 2007 version and Table S4). PCR amplification was performed with the following parameters: the PCR mixture contained 10 ng bisulfite-treated DNA, 200 mM dNTPs, 0.2 U of Hot Start Taq DNA polymerase (QIAGEN), and 0.2 mM forward and reverse primers in a total volume of 5µl. The cycles included a hot start at 94°C for 15 min, followed by denaturation at 94°C for 20 sec, annealing at 56°C for 30 sec, extension at 72°C for 1 min (45 cycles), with a final incubation at 72°C for 3 min. Unincorporated dNTPs were dephosphorylated by adding 2 ml of premix including 0.3 U shrimp alkaline phosphate (SAP; Sequenom). The reaction mixture was incubated at 37°C for 40 min and SAP was then heat inactivated for 5 min at 85°C. After SAP treatment, 2 ml of the PCR product was used as a template for *in vitro* transcription and RNase A cleavage was used for the reverse reaction, per the manufacturer’s instructions (Sequenom). The samples were conditioned and spotted on a 384-pad Spectro-CHIP (Sequenom) using a MassARRAY nanodispenser (Samsung, Irvine, CA, USA), followed by spectral acquisition on a MassARRAY analyzer compact MALDI-TOF mass spectrometer (Sequenom). The methylation analyses were carried out using the EpiTYPER application (Sequenom) to generate quantitative results for each CpG site or an aggregate of multiple CpG sites.

### Determination of HPV Genotypes

Genomic DNA was extracted from cervical tissues using a QIAamp DNA Mini Kit?(QIAGEN, Dusseldorf, Germany) according to manufacturer’s instructions. The concentration and purity of the DNA were determined by absorbance at 260 and 280 nm. HPV genotypes were detected using Human Papillomavirus Genotyping Diagnosis Kit (yjkuang Genetel Pharmaceuticals, Shenzhen, China)and analyzed using HPV genotypes DNA microarray reader system(HPV-GenoCam-9600, yjkuang Genetel Pharmaceuticals, Shenzhen, China))as described [24]. Briefly, HPV DNA was extracted and PCR amplified using the following parameters: denaturation at 95°C for 10 min, followed by 40 cycles of 95°C for 30 seconds, 52°C for 45 sec, and 65°C for 90 sec. At the end of the last cycle, the mixture was incubated at 65°C for 5 min. The gene chip was prepared, PCR products hybridized, and visualized.

### Statistical Analyses

All statistical analyses were performed with the SPSS Version 17 software package. All P values were two-sided and the significance level was *P*<0.05. Mann-Whitney test were used to test continuous variables for differences in immunohistochemical staining scores between tumor and normal tissues for the HLA-I and APM components. Fisher’s exact test was used for evaluation of associations with clinical pathological parameters. Quantitative DNA methylation data derived from MassARRAY were treated as continuous variables and missing measurements were imputed into multivariable regression analyses using samples with replacement for the nonmissing values (single imputations). Linear associations between 2 continuous variables were quantified by Pearson correlation coefficient.

## Results

### APM Component and HLA- I Expression in Cervical Lesions

**Figure 1:**
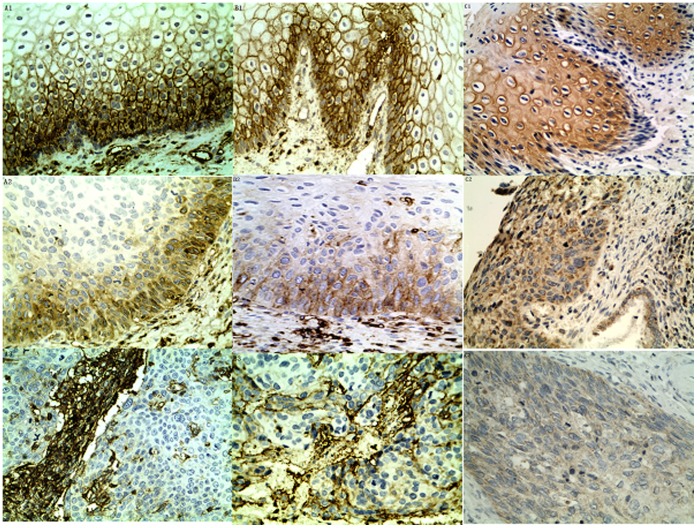
Immunohistochemical analysis. A-C. Staining patterns of HLA-I, ß2-m and ERAP1, respectively. (Other APM components TAP1, TAP2, LMP2, LMP7, calnexin, calreticulin, ERp57, and tapasin staining shown in Figure S1), Panels A1–C1 depict normal uterine cervix tissue with normal protein expression. Panels A2-C2 show cervical intraepithelial neoplasia with partial loss of protein expression. Panels A3-C3 show cervical carcinoma with weak or total loss of protein expression.

All the antibodies were tested on formalin-fixed, paraffin-embedded, normal cervical tissues, CIN and CSCC. Representative staining patterns for the HLA class I heavy chains, ß2-m and APM components are shown in Figs. 1 and Figure S1. As shown in Figure 1, Staining of HLA class I heavy chains and ß2-m were localized on the cell membranes of the cervical epithelium and interstitial cells, whereas the APM components staining were mainly localized in the cytoplasm of cervical epithelium. Anti- HLA class I and anti- ß2-m antibodies showed strong staining of cell membranes in normal cervical tissue, whereas a highly variable expression profile in CIN and CSCC tissue samples, ranging from partial loss to total loss of expression, but decreased expression, to normal expression. Expression of the HLA class I heavy chains, ß2-m and APM components are summarized in Table 1.

**Table 1:** Heterogeneous expression of HLA-I and APM components in cervical lesions.

	Normal control (n = 25)			*P*	CIN (n = 64)			*P*	CSCC (n = 63)			*P*
	Total loss	Partial loss	Normal expression		Total loss	Partial loss	Normal expression		Total loss	Partial loss	Normal expression	
HLA-I	1 (4.0)	1 (4.0)	23 (92.0)	<0.001	19 (29.7)	20 (31.3)	25 (39.1)	<0.001	30 (47.6)	20 (31.7)	13 (20.6)	<0.001
ß2-m	1 (4.0)	1 (4.0)	23 (92.0)	<0.001	15(23.4)	19(29.7)	30(46.9)	<0.001	25(39.7)	20(31.7)	18(28.6)	<0.001
TAP1	1 (4.0)	3 (12.0)	21 (84.0)	0.006	13 (20.3)	23 (35.9)	28 (43.6)	0.006	23 (36.5)	20 (31.7)	20 (31.7)	0.006
TAP2	1 (4.0)	1 (4.0)	23 (92.0)	0.001	15 (23.4)	18 (28.1)	31 (48.4)	0.012	25 (39.7)	20 (31.7)	18 (28.6)	0.001
LMP2	0	2 (8.0)	23 (92.0)	0.001	14 (21.9)	20 (31.3)	30 (46.9)	0.001	18 (28.6)	23 (36.5)	22 (34.9)	0.039
LMP7	1 (4.0)	3 (12.0)	21 (84.0)	0.004	13 (20.3)	23 (35.9)	28 (43.6)	0.005	22 (34.9)	21 (33.3)	20 (31.7)	0.004
ERAP1	1 (4.0)	1 (4.0)	23 (92.0)	0.02	14 (21.9)	19 (29.7)	31 (48.4)	0.02	13 (20.6)	20 (31.7)	30 (47.6)	0.02
tapasin	2 (8.0)	3 (12.0)	20 (80.0)	0.039	10 (15.6)	18 (28.1)	36 (56.3)	0.031	18 (28.6)	15 (23.8)	30 (47.6)	0.039
calnexin	1 (4.0)	1 (4.0)	23 (92.0)	0.058	10 (15.6)	14 (21.9)	40 (62.5)	0.042	22 (34.9)	13 (20.6)	28 (44.4)	0.068
calreticulin	2 (8.0)	3 (12.0)	20 (80.0)	0.041	14 (21.9)	10 (15.6)	40 (62.5)	0.048	17 (27.0)	14 (22.2)	32 (50.8)	0.051
ERp57	2 (8.0)	2 (8.0)	21 (84.0)	0.018	14 (21.9)	13 (20.3)	37 (57.8)	0.028	18 (28.6)	17 (27.0)	28 (44.4)	0.004

**Figure 2:**
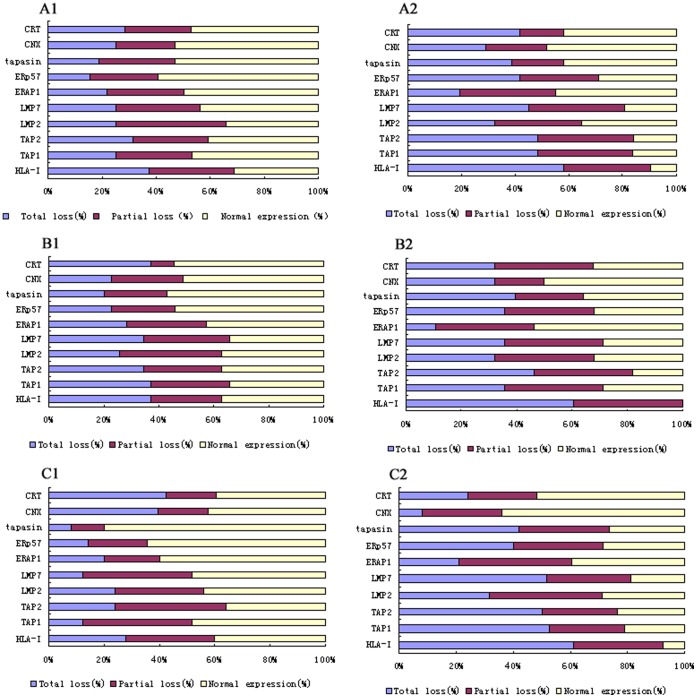
Down-regulation of HLA-I and APM components during the malignant process of cervical cancer. Analyze the clinicopathologic characteristics (tumor grad, clinical stage and Lymph node metastases) with protein expression of HLA-I and APM components (TAP-1, TAP-2, LMP2, LMP7, Tapasin, ERAP1, CNX, CRT and ERp57) in cervical cancer.Results are represented as percentage (%). upper histogram A1 show HLA-I and APM components expressed in well differentiated tumor, A2 show expressed in Moderate to poorly differentiated tumor. B1 show expressed in ≤II a tumor and B2 show expressed in ≥II B, C1 show expressed in Lymph node metastases negative tumor and C2 show expressed in Lymph node metastases positive tumor, respectively. * *P* < 0.05.

Of the 64 cases CIN and the 63 cases CSCC enrolled in this study (Table I and Figure 2), the protein expression level of HLA class I and APM components was altered from normal expression to partial loss or total loss, with the development of normal epithelium of uterine cervix to CIN and CSCC. The total loss of protein expression was remarkable for most of the members of HLA class I and APM family in CSCC compared with CIN or normal controls, except for CNX and CRT. The partial loss of protein expression of HLA-I, ß2-m, TAP1, TAP2, LMP2, LMP7, ERAP1, tapasin and ERp57 was statistically significant for CIN cases compared to normal controls.

The association between the expression of HLA class I, ß2-m, and APM components and clinicopathological characteristics was investigated in CSCC lesions. As can be seen in Figure 2A, there was an inverse correlation with downregulation of HLA-I, ß2-m TAP1, LMP7, and ERp57 protein expression and tumor differentiation grade. Using FIGO staging criteria, ERp57 protein expression was significantly lower in lower stage tumors than in higher stages (Figure 2B). In addition, downregulation of HLA-I, TAP1, LMP7, tapasin, CRT, and ERp57 had an inverse correlation with lymph node metastasis (Figure 2C). Both correlations were statistically significant (P<0.05). The downregulation of ß2-m,TAP1, TAP2, LMP7, ERAP1, tapasin, and ERp57 protein expression positively associated with HLA-I expression in CIN lesions, whereas ß2-m, TAP1, TAP2, LMP2, LMP7, ERAP1, ERp57, and tapasin expression positively associated with HLA-I in the cancerous lesions. Down-regulation of any APM component except calnexin and calreticulin was significantly associated with HLA class I down-regulation in CIN and CSCC group (P<0.05).

**Figure 3:**
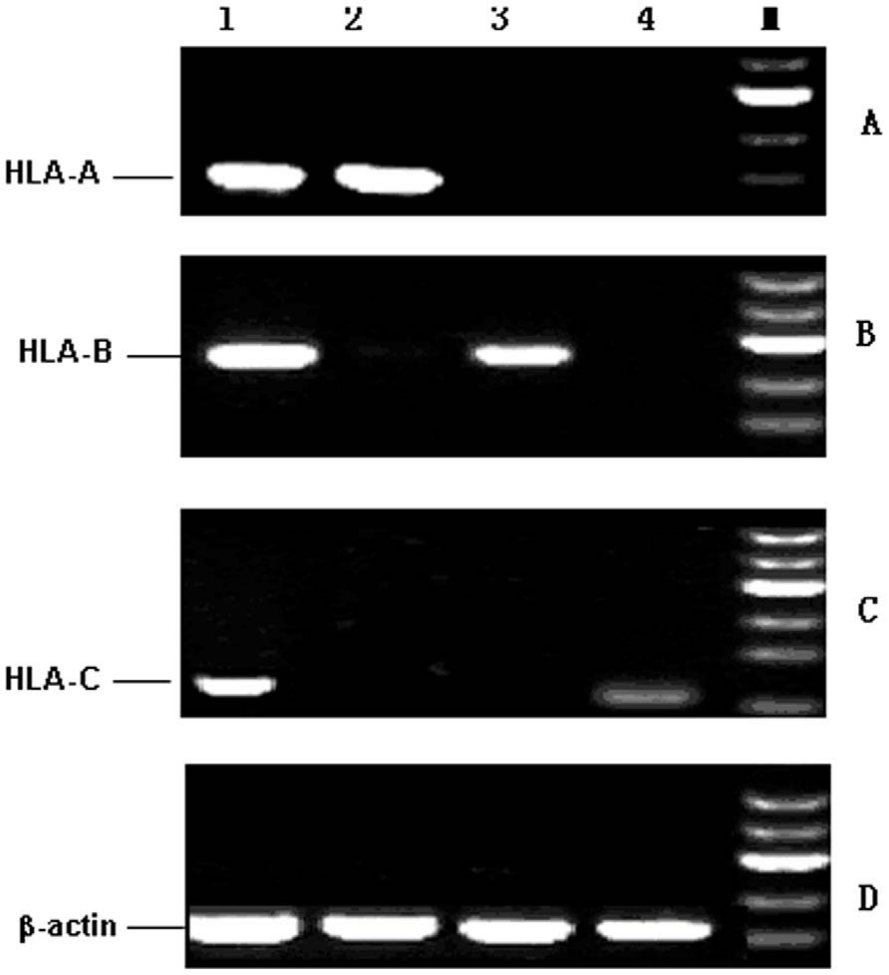
mRNA expression patterns of HLA-A, HLA-B and HLA-C (A–C). M: 100–600 bp marker ladders. Lanes 1 show normal cervical tissue, 2 and 3 shows CIN, 4 shows CSCC tissue. mRNA levels were normalized to β-actin mRNA (D). The lack mRNA expression of a single allele of HLA-I will affect the normal surface expression of HLA-I molecules according to protein detected by IHC.

**Figure 4:**
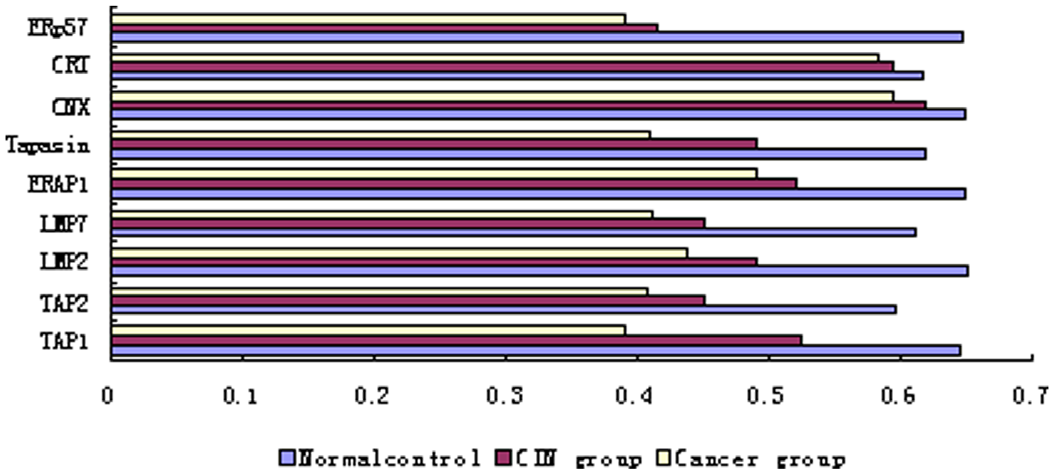
Down-regulation of APM genes during the progression of cervical lesions. Expression levels of the APM components (TAP-1, TAP-2, LMP2, LMP7, Tapasin, ERAP1, CNX, CRT and ERp57) were analyzed by Semi-quantitative RT-PCR in normal uterine cervix tissue, CIN and CSCC. Compared to the control, CIN and CSCC, mRNA levels were normalized to β-actin mRNA. The upper histogram has shown the expression levels of the APM components in in normal uterine cervix tissue, CIN and CSCC. The results of RT-PCR are represented as means ± SD from 15 controls, 30 CIN and 33 CSCC respectively. * *P* < 0.05.

To further verify the results of immunohistochemical staining analysis, we detected the transcription of HLA class I and APM components by semi-quantitative RT-PCR. The expression level of HLA-A,-B and -C coding for HLA-I was lower in both CIN and CSCC than in controls (Figure 3). The transcripts of TAP-1/2 and LMP2/7 were hard to detect, and no transcription of Tapasin and ERp57, in CIN and CSCC tissues. However, no difference was found for CNX and CRT transcription between CIN or CSCC and controls (Figure 4). Although the samples for RT-PCR and IHC analysis were from different origin, these results suggested that the trend of downregulation of target gene expression at both transcriptional and translational level was comparable in the development of cervical cancer, especially in the pathogenesis of precursor lesions.

### Methylation Profiles of the APM Gene Family in a CSCC Cell Line

**Figure 5:**
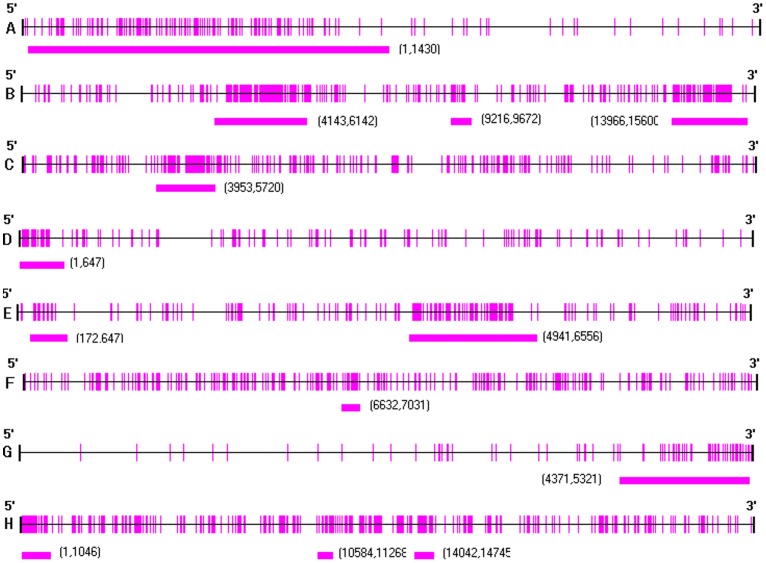
The CpG sites analyzed in promoters of APM genes family. Each vertical indicate an individual CpG site. Solid line positions of BSP primers. All the BSP primers are designed to cover the transcriptional start site should be close to transcriptional start site. Panels A-H depict HLA-B, TAP-1, TAP-2, LMP2, LMP7, Tapasin, ERAP1 and ERp57.

Because of the theoretical relationship between gene promoter hypermethylation as an epigenetic modification with the downregulation of gene transcription, we analyzed HLA-B, TAP1, TAP2, LMP2, LMP7, ERAP1, tapasin, and ERp57, due to the downregulation of these genes in cervical cancer or precursor lesions, for methylation status of CpG islands at the promoter region in SiHa cervical carcinoma cells by bisulfite-sequencing approach. By amplification of target CpG islands identified by professional software with bisulfate sequencing primers (BSP, Figure 5) using genomic DNA as template, and followed by cloning and sequencing, we found various extents of CpG site methylation of TAP1, TAP2, LMP7, tapasin and ERp57 genes. However, no methylation was detected for HLA-B, LMP2, and ERAP1. All of the CpG sites of methylated candidate genes are shown in Table S4,in which the location of primers were presented in underlines, target CpG sites in italics and methylated CpGs in bold.

### DNA Methylation in Cervical Lesions and its Association with mRNA Expression and HPV Infection

By an approach using Sequenom MassARRAY platform for quantitative detection of methylated DNAs, we found that the global methylation level of target CpG islands of TAP1, LMP7 and ERp57 genes was significantly higher in genomic DNA of CSCC than in either CIN or normal controls (*P<*0.05), but no difference was found for TAP2 and tapasin (Table 2). The methylation level of TAP1 was significantly higher in both CIN and CSCC than in controls (*P* = 0.015 and 0.001, respectively). Of LMP7 and ERp57 genes, the methylation level in CSCC, but not in CIN, significantly different from the control (*P = *0.001). Further analysis of single CpG site specific methylation within TAP1, LMP7, and ERp57 indicated a significant increase in methylation level of most of the CpG sites at target CpG islands of TAP1, LMP7, and ERp57 genes in CSCC compared to controls, whereas no statistical significance was found between CIN and the control (*P*>0.05).

**Table 2:** Methylation levels of TAP1, TAP2, LMP7, tapasin and ERp57 in CIN and CSCC samples.

Group	TAP1	TAP2	LMP7	Tapasin	ERp57
Control	0.0345±0.0291	0.8578±0.1573	0.0652±0.0488	0.9285±0.0765	0.8040±0.1705
CIN	0.0370±0.0261	0.8490±0.1674	0.0728±0.0548	0.9298±0.0794	0.8697±0.1336
CSCC	0.0477±0.0387	0.8399±0.1599	0.1864±0.2893	0.9194±0.0888	0.9065±0.0709
*P*	*P* = 0.015, 0.001	*P* = 0.704	*P* = 0.0001	*P* = 0.341	*P* = 0.0001

Further analysis of the data resulted from the quantitative analysis of single CpG site methylation by Sequenom MassARRAY platform, has shown that TAP1 gene contains 23 CpG sites and the methylation level between two or three CpG sites from CpG_1, CpG_2.3, CpG_4, CpG_16, CpG_19, CpG_20, CpG_21, CpG_22 and CpG_23 associated with each other, but no association was found for the methylation ratio of other CpG sites. LMP7 gene contains 22 CpG sites and the methylation level between CpG_1, CpG_2, CpG_6, CpG_8, CpG_9, CpG_12, 13, 14, CpG_20 and CpG_22 associated with each other. ERp57 gene contains 9 CpG sites, in which CpG_1, CpG_2, CpG_5, CpG_6, CpG_7 have significance between control and cancer group. Although no statistical significance were found of whole target CpG fragment methylation level of TAP2 between three groups, the analysis of the data resulted from the quantitative analysis of TAP2 single CpG site methylation has shown that there was significant differences in the methylation level of CpG_8 sites between CSCC and controls, but no association was found for the methylation ratio of other CpG sites (*P*<0.05).

In addition, the comparative analysis of TAP1, LMP7, and ERp57 methylation with the protein expression levels and high-risk HPV16 infection showed an inverse correlation of altered CpG island methylation with changes in protein expression of corresponding genes. Moreover, the methylation level of these genes was significantly higher in cases positive for HPV16 infection than in negative ones (Tables 3 and 4).

**Table 3:** Inverse correlation between methylation and protein expression in cervical lesions.

Protein expression	Methylation levels ( 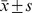 )					
	TAPI	F *P*	LMP7	F *P*	ERp57	F *P*
−	0.0357±0.0298	8.750.038	0.1732±0.0558	8.690.035	0.7203±0.1730	8.960.038
+	0.0325±0.0271		0.0864±0.0793		0.7032±0.1227	
++	0.0275±0.0201		0.0528±0.0048		0.6527±0.1102	
+++	0.0245±0.0199		0.012±0.0013		0.6024±0.1319	

**Table 4:** APM promoter methylation is higher in patients with HPV16 infection.

HPV16 status	global methylation level of target genes ( 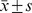 )				
	TAP1	TAP2	LMP7	Tapasin	ERp57
positive	0.0483±0.0179	0.8715±0.15602	0.0760±0.0617	0.9322±0.08065	0.8751±0.12923
negative	0.0392±0.0319	0.8639±0.17738	0.0670±0.0572	0.9275±0.08841	0.7240±0.16017
*P*	9.08	1.823	8.583	0.458	12.908
	0.035	0.734	0.0491	0.634	0.001

## Discussion

Reduced immunocompetence associated with the downregulation of HLA-I expression has been frequently reported for patients with increased tumor invasion and metastasis, implying that the functional abnormality of HLA-I in cellular antigen presentation most probably be a key event in tumor development and progression [20], [25]–[26].

In this study, we investigated the association of cervical carcinogenesis and the pathogenesis of its precursor lesions with aberrant regulation of HLA-I and APM expression at different levels including gene promoter methylation, transcription and protein expression. The results have shown that the development of normal epithelium of uterine cervix to CIN and CSCC was accompanied with altered protein expression of HLA-I, TAP1, TAP2, LMP2, LMP7, ERAP1, tapasin and ERp57 from normal expression to partial loss or total loss of expression in cervical tissue specimens in Immunohistochemical analysis. This was positively correlated with the downregulation of corresponding gene transcription, in addition to HLA-A, HLA-B and HLA-C genes coding for HLA-I, in fresh cervical lesions detected by semi-quantitative RT-PCR. But, due to the limited potential of HLA-I antibody (in this case, the antibody HC-10) in recognizing the heavy chains, especially the heavy chain of HLA-A, the positive signals of HLA-A expression may most probably be lost in IHC analysis, and therefore the conclusion above was to some extent also limited. Thus, future studies are needed to verify the results of this study with other HLA-I specific antibodies.

Thus, the transcriptional downregulation and the loss of protein expression of HLA-I and other APM family members may be involved in the development of cervical cancer in Uighur women. This was in consistence with previous reports that HLA-I and APM protein expression was partially and totally lost in cervical cancer specimens and associated with its clinical pathological outcome [20]. However, the relationship between the regulation of HLA-I and APM expression and the pathological state of cervical precursor lesions, i.e. the development of CIN, described in this study has not been reported so far. Most of the previous studies demonstrated the deregulation of APM gene expression in different kinds of malignant tumors at protein level, but the aberrant regulation of these genes at transcription level was not described so far [27]–[29]. It is noteworthy that abnormalities in APM gene expression, mainly the downregulation of TAP1, TAP2, LMP7, ERAP1, tapasin, and ERp57 expression, associated with HLA-I in cervical lesions may attribute to deregulation of HLA-I mediated antigen presentation, and the understanding of an immune escape mechanism of tumor cells in cervical carcinogenesis.

Downregulation of HLA-I antigen in tumor cells is believed to allow immune escape from the host and thereby allows tumor cells to develop a more aggressive phenotype [30]. Indeed, among the clinicopathological parameters we examined, abnormalities of HLA-I surface expression attributable to APM deficiencies was significantly associated with the metastasis of CSCC to lymph nodes. Several previous studies also showed that HLA-I molecules and APM components were downregulated in others cancers and associated with poor prognoses [31]–[32]. In our previous report [33], the number of infiltrating CD8^+^ T cells inside tumor lesions was dependent on HLA-I expression in tumor lesions, an observation also seen in esophageal carcinomas [27]. Therefore, we hypothesize that the advanced clinicopathological features in CSCC patients with downregulated HLA-I expression might be due to reduced infiltration of CD8^+^ T cells, which provides tumor cells with an escape from T lymphocyte-mediated cytotoxicity.

A might mechanism of altered gene expression level was epigenetic modification and one of the most common mechanisms that produce epigenetic changes is DNA methylation. In this study, we using bisulfite sequencing analysis identified high level CpG island methylation of TAP1, TAP2, LMP7, tapasin, and ERp57 gene promoters. However, no methylation was detected for HLA-B, LMP2, and ERAP1. However, no difference in methylation was found for TAP2 or tapasin. It is noteworthy that epigenetic modifications may not always occur in genes coding for HLA-I heavy chain with the result of altered gene expression. Further quantitative DNA analysis of the CpG islands indicated significantly higher methylation for TAP1, LMP7, and ERp57 genes in CSCC tissue as compared to CIN and normal tissues. Additional analyses showed an inverse correlation of altered TAP1, LMP7, and ERp57 CpG island methylation with changes in protein expression.

In previous studies, gene promoter hypermethylation was associated with downregulation of tapasin, TAP1, TAP2, and LMP7 protein expression in esophageal squamous cell carcinoma, colon cancer, renal cancer, and melanoma. These effects were reversible upon treatment with methyltransferase inhibitors, providing evidence that APM gene expression is regulated by methylation [34]. Altered chromatin structure of HLA-I heavy chain or methylation of HLA-A, B, and C at promoter regions is an important mechanism of transcriptional inactivation in esophageal squamous cell cancer and primary and metastatic melanoma [35]. Jasper et al. reported upregulated protein expression of TAP1 and TAP2 in SiHa cervical cancer cells with treatment of methyltransferase inhibitors, providing additional evidence for the inhibitory effect of methylation on gene expression [36].

The quantitative analysis of methylated DNA by Sequenom MassARRAY platform, a mass spectrometry approach, is based on the detection of methylation state of a single CpG site at a target fragment (CpG island) and generates a data representing the ratio or frequency of the methylation events on a CpG site in all DNA molecules, i.e. DNA templates, of a certain sample. Because of the enzyme- specific cleavage of target fragments before mass spectrometric detection, the methylation level (ratio) of a certain CpG site is independent, to some extent, from the other CpG sites located at the same target fragment. Thus, the data provided by mass spectrometric detection has to be further analyzed for the association of different CpG sites at methylation level to evaluate the methylation state of whole target fragment (CpG island). In this study, we have shown that the alteration of methylation ratio at all single CpG sites of the target fragment of TAP1,LMP7 and ERp57 genes was positively correlated, and the methylation level of these CpG sites was significantly higher in CIN or CSCC cases compared with normal. Therefore, the overall methylating level can not represent the clinical value. In fact, only the accurate information of CpG sits methylating level represent the clinical application value.

In the present study, we also found that the promoter methylation of TAP1, LMP7, and ERp57 genes was significantly higher in cases positive for human papillomavirus 16 (HPV 16) than negative ones. Aberrant CpG island methylation of these genes promoter in cervical cancer cell may reveal an epigenetic regulation of gene expression caused HPV16 infection. Despite the great advances made in understanding human papillomavirus structure and the molecular mechanisms of cervical cancer development in recent years, many details of how the HPV escapes host immunity are yet to be understood. The persistence of high-risk HPV is the greatest risk factor for the development of cervical cancer. However, to persist, HPV must developed specific strategies to escape the host immune system or evolved intricate ways to regulate and evade the host’s immune defense, which include that viruses employ to imitate the host genome and undergo latency to evade the host’s recognition of the pathogen, they have also developed epigenetic mechanisms by which they can render the host’s immune responses inactive or silence to their antigens, that is epigenetic regulation of immune gene.

Several important reports revealed a novel strategy on how high-risk HPVs can abolish the innate immune response in their genuine host cells in cervical cancer were demonstrated, in which an epigenetic silencing of type I IFN after HPV16 oncogene expression and that IFN-κ expression is suppressed by de novo methylation within a CpG-rich area near the transcriptional start site, IFN-κ being the only keratinocyte-specific IFN involved in innate immunity. Inhibition of this pathway may represent an early and central event in the development of cervical cancer [37]–[38]. As a potential mechanism for pathogenic infection and gene methylation, it was also hypothesized that the pathogenic infection causes a host inflammatory reaction followed by nitric oxide release to activate methyltransferases, which leads to gene methylation [39]. It is evident that the gene promoter methylation of RAS family genes in cervical cancer tissues is associated with HPV infection but the exact mechanism for methylation by HPV remains unclear [40].

Although persistent infection of HPV is the greatest risk factor for the development cervical cancer, progression to cancer requires more than just HPV infection and persistence. Our study provides epigenetic evidence that HPV16 related epigenetic modifications is important factors allowing the tumor cell to escape immune surveillance and also demonstrate that deficiency of surface HLA-I is caused indirectly by epigenetic silencing of APM genes in cervical lesions in Uighur women. Therefore, further investigations of the host-HPV relationship may provide a deeper understanding of the nature of HPV-resistance to host immunity and HPV-related gene silence as well as the development of HPV-induced cancers, which will useful for understanding the primary mechanism of APM epigenetic regulation is critical and would be clinically and therapeutically important.

## Supporting Information

Figure S1 Immunohistochemical analysis: D-L. Staining patterns of TAP1, TAP2, LMP2, LMP7 and calnexin, calreticulin, ERp57, and tapasin, respectively. Panels D1–L1 depict normal uterine cervix tissue with normal protein expression. Panels D2-L2 show cervical intraepithelial neoplasia with partial loss of protein expression. Panels D3-L3 show cervical carcinoma with weak or total loss of protein expression.
(TIF)

Table S1 Primer sets of the target genes.
(DOC)

Table S2 Bisulfite-sequencing PCR (BSP) primer sets.
(DOC)

Table S3 Methylation-specific primers of the target genes.
(DOC)

Table S4 The CpG methylation sites in target gene promoter fragments.
(DOC)
