# Supplementary figures and images for: Post-Transcriptional and Epigenetic Regulation of Antigen Processing Machinery (APM) Components and HLA-I in Cervical Cancers from Uighur Women
Source: PLoS One. 2012 Sep 14;7(9):e44952. doi: 10.1371/journal.pone.0044952 (PMC3443204; doi:10.1371/journal.pone.0044952)

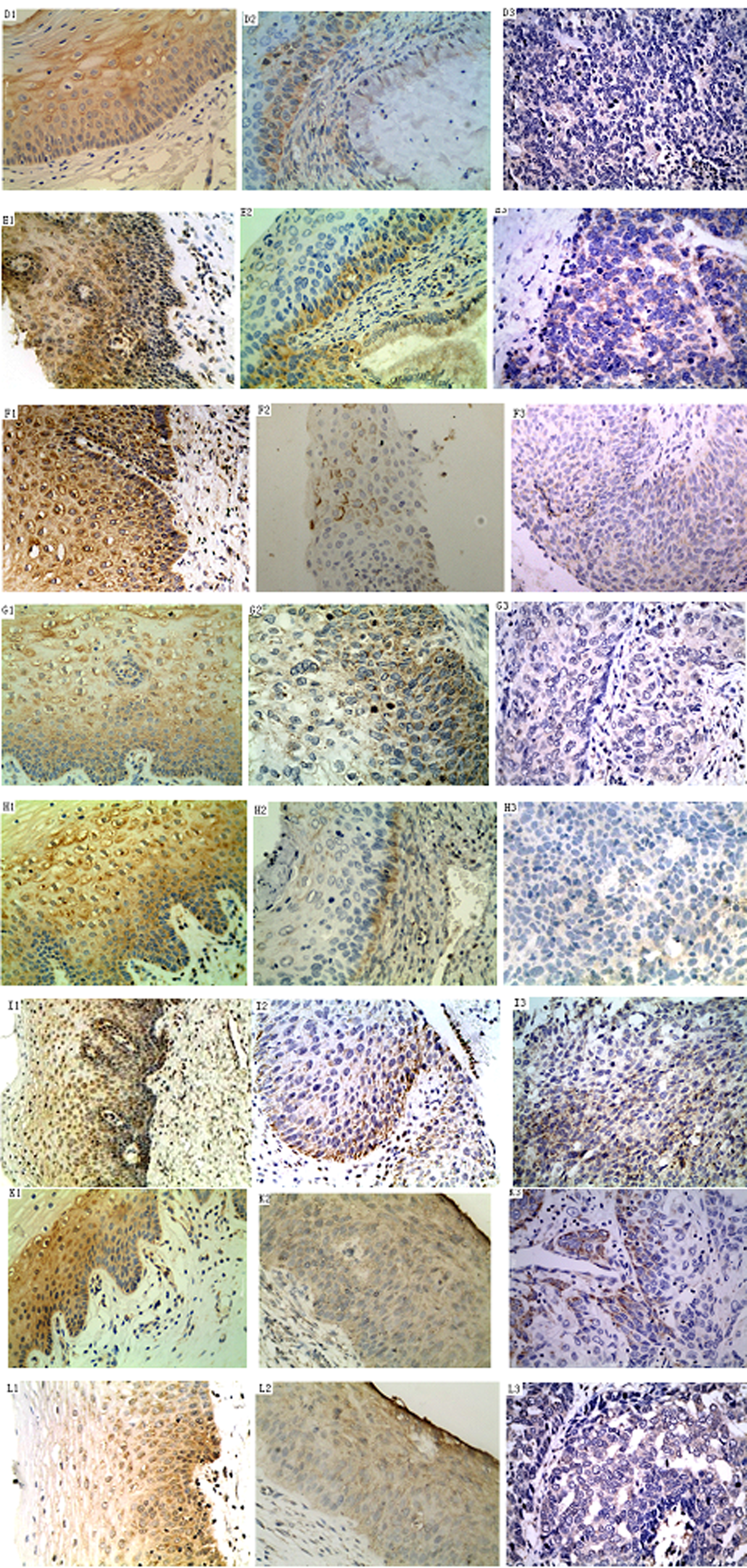

Supplement: Figure S1 — Immunohistochemical analysis: D-L. Staining patterns of TAP1, TAP2, LMP2, LMP7 and calnexin, calreticulin, ERp57, and tapasin, respectively. Panels D1–L1 depict normal uterine cervix tissue with normal protein expression. Panels D2-L2 show cervical intraepithelial neoplasia with partial loss of protein expression. Panels D3-L3 show cervical carcinoma with weak or total loss of protein expression. (TIF) [file pone.0044952.s001.tif]
