# Supplementary material for: Post-Transcriptional and Epigenetic Regulation of Antigen Processing Machinery (APM) Components and HLA-I in Cervical Cancers from Uighur Women
Source: PLoS One. 2012 Sep 14;7(9):e44952. doi: 10.1371/journal.pone.0044952 (PMC3443204; doi:10.1371/journal.pone.0044952)
Supplement: Table S1 — Primer sets of the target genes. (DOC) [file pone.0044952.s002.doc]

**Table S1**

| Gene | Primer（5'-3'） | Product |
| --- | --- | --- |
| HLA-A | 5'TCCTTGGAGCTGTGATCGCT 3' | 274 bp |
|  | 5'AAGGGCAGGAACAACTCTTG 3' |  |
| HLA-B | 5'ATTACATCGCCCTGAACGAG 3' | 151 bp |
|  | 5'ATCTCCGCAGGGTAGAAACC 3' |  |
| HLA-C | 5'TCCTGGTTGTCCTAGCTGTC 3' | 456 bp |
|  | 5'CAGGCTTTACAAGTGATGAG 3' |  |
| Calnexin | 5'GGCTAGACGACGAACCTGAG 3' | 188 bp |
|  | 5'AGGCTTCCATTTGCCCTTAT 3' |  |
| Calreticulin | 5'AGGCTCCTTGGAGGATGATT 3' | 207 bp |
|  | 5'TCCCACTCTCCATCCATCTC 3' |  |
| TAP1 | 5'GACAAGAGCCGCTGCTATTTGG 3' | 345 bp |
|  | 5'TGATAAGAAGAACCGTCCGAGA 3 |  |
| TAP2 | 5'GCCTGTGCTGTTCTCGGGTTCTGC 3' | 441 bp |
|  | 5'TGTACCAGGTGGGCGTAG 3' |  |
| LMP2 | 5'CTCTGCACCAGCACATCTT 3' | 376 bp |
|  | 5'AGAGTGATGGCATCTGTGGT 3' |  |
| LMP7 | 5'ATGGCGTTACTGGATCTGTGCGGTGC 3 | 482 bp |
|  | 5'TCACAGAGCGGCCTCTCCGTACTTGTA 3 |  |
| Tapasin | 5'GAAAAGTAAGGTTAGGTGTGGT 3' | 273 bp |
|  | 5'CTTATTACCCAAACTAAAATACCA 3' |  |
| ERp57 | 5'GAGGTAGGGAGTTTAGTTTAGG 3' | 259 bp |
|  | 5'TAACTTCTCTAAATATCCAATACCCA 3' |  |
| ERAP1 | 5’GGCCTGGCAATTTCTGAGGA 3' | 124 bp |
|  | 5’TTCAAGCCGTGTTCTTGTGGAG 3' |  |
| β-actin | 5’TCACCCACACTGTGCCCATCT 3’ | 106 bp |
|  | 5’GTGAGGATCTTCATGAGGTAGTCAGTC 3’ |  |
