# Supplementary material for: Post-Transcriptional and Epigenetic Regulation of Antigen Processing Machinery (APM) Components and HLA-I in Cervical Cancers from Uighur Women
Source: PLoS One. 2012 Sep 14;7(9):e44952. doi: 10.1371/journal.pone.0044952 (PMC3443204; doi:10.1371/journal.pone.0044952)
Supplement: Table S2 — Bisulfite-sequencing PCR (BSP) primer sets. (DOC) [file pone.0044952.s003.doc]

**Table S2**

| Target gene | Primer（5'-3'） | Product |
| --- | --- | --- |
| HLA-B | Forward GAGGTAGGGAGTTTAGTTTAGG | 370 bp |
|  | Reverse TGGGTATTGGATATTTAGAGAAGTTA |  |
| TAP1 | Forward GGTATTGGTTTTTAATTTGGGA | 331 bp |
|  | Reverse TGGGTAGGTTATTTTTGGAAGT |  |
| TAP2 | Forward AAGGTTTTGGGTTAGGAAGG | 259 bp |
|  | Reverse GAAAATTTTTTTTGGTTTGGTG |  |
| LMP2 | Forward TGTCCCAGGTTGGAAACCAGTGCC | 443bp |
|  | Reverse TAGTGGGGGTTGGTTAAATTA |  |
| LMP7 | Forward TGTGATGGTTTTGGTTTAGGTA | 334 bp |
|  | Reverse GAAGTTTTTAGGGATGTAGGGAG |  |
| Tapasin | Forward GAAAAGTAAGGTTAGGTGTGGT | 273 bp |
|  | Reverse TGGTATTTTAGTTTGGGTAATAAG |  |
| ERp57 | Forward AAGATTTAGGGTTTTTTGAAAT | 259 bp |
|  | Reverse GGAGGTAGAGTTTGTAGTGA |  |
| ERAP1 | Forward AATTTTTGGGGTATAGTGGTTTT | 133 bp |
|  | Reverse GTTTAGGTTTGGTGGATTTGTTA |  |
