# Supplementary material for: Post-Transcriptional and Epigenetic Regulation of Antigen Processing Machinery (APM) Components and HLA-I in Cervical Cancers from Uighur Women
Source: PLoS One. 2012 Sep 14;7(9):e44952. doi: 10.1371/journal.pone.0044952 (PMC3443204; doi:10.1371/journal.pone.0044952)
Supplement: Table S3 — Methylation-specific primers of the target genes. (DOC) [file pone.0044952.s004.doc]

**Table S3**

| Target gene | Primer（5'-3'） | Product |
| --- | --- | --- |
| TAP1 | aggaagagagGTTTGGGGTATTGGTTTTTAATTTG | 331 bp |
|  | cagtaatacgactcactatagggagaaggctCCTACTTCCAAAAATAACCTACCCA |  |
| TAP2 | aggaagagagGTATAGGAAGGTTTTGGGTTAGGAA | 259 bp |
|  | cagtaatacgactcactatagggagaaggctAAATACCCTCTTCCATACAAACTCC |  |
| LMP7 | aggaagagagTTAGTGTGATGGTTTTGGTTTAGGT | 334 bp |
|  | cagtaatacgactcactatagggagaaggctATCCCTAAAAACTTCCCTACTACCC |  |
| Tapasin | aggaagagagTAGGTAAGAAAATGAAAAGTAAGGTTAGG | 273 bp |
|  | cagtaatacgactcactatagggagaaggctCTCTTATTACCCAAACTAAAATACCA |  |
| ERp57 | aggaagagagTTTAGGGTTTTTTGAAATAAAAGGGGT | 259 bp |
|  | cagtaatacgactcactatagggagaaggctACCAAAATAATCTCTATCTCCTAACCTC |  |
