# Supplementary material for: Post-Transcriptional and Epigenetic Regulation of Antigen Processing Machinery (APM) Components and HLA-I in Cervical Cancers from Uighur Women
Source: PLoS One. 2012 Sep 14;7(9):e44952. doi: 10.1371/journal.pone.0044952 (PMC3443204; doi:10.1371/journal.pone.0044952)
Supplement: Table S4 — The CpG methylation sites in target gene promoter fragments. (DOC) [file pone.0044952.s005.doc]

**Table S4**

| Target gene | target gene promoter fragments | product | positions of BSP primers |
| --- | --- | --- | --- |
| TAP1 | GGTATTGGTTTTTAATTTGGGATAG*CG*TATAA***CG****CG*TAGT***CG***ATAGTT  T*CG*TTTTTT*CGCG*GCGT*CG*TTAGGAGGCGTTTGGGTGTTG*CG*GGGTTG  TTTTG*CGCGCG*G*CG*TTAA*CG*TGTGTAGGGTAGATTTGTTT***CG***AGATAA  GTGA*CG*AGGTAGTTT***CG***TTTTGAGGTTGGGGTGGGAAAATTGGTGTAA  GTGGAAAGGTAGGAGGTAGGGAGAGG*CG*AGAAGGGTGTG***CG***TGATGGA  GAAAATTGGGTATTAGGGTTGTTTT*CG*AGATTTTTAGATTTGATTTTT  A*CG*TTTGTTATTAAAATAGTTTGGGTAGGTTATTTTTGGAAGT | 330 bp | -226～+104 |
| TAP2 | AAGGTTTTGGGTTAGGAAGGATATTTAAAAATTTAATTATTGTTTTA  AATAGTGTTAAAATAGAATAAGAATTAAAGTTTAGTA***CG***GGGTATTT  TTTTTATAGGTTGAAGGTG***CG***TTTAATATAATTTGGAGTATAGATTT  AGAGGTATTTGAATA***CGCG***TTAGTTTAAGGTGTTT***CG***GTTGAGAAGG  A***CG***GATGAAGATGAA***CG***TTTAGGGTTTATTAAATTTAAAGTTTGTA***CG***  TGAAAATTTTTTTTGGTTTGGTG | 259bp | -725～-984 |
| LMP7 | TGTGATGGTTTTGGTTTAGGTATTAATTGTTTTTTTTT*CG*GAAAAGGT  AGGGGGATGTGGAAAAGAGTTTTGTTTTTTTTTTTT*CG*ATTTGTGGTT  TT*CG*TTTTTATTTTTTTTTT*CG*AGAG*CG*GATAGATTTTTGGGTGTTGGG  CGGTTATGG*CG*TTATTAGATGTATG*CG*GAGTTTTT*CG*AGGGTAG*CG*GT  *CG*GAAT*CG*GTTTTTT*CG*GTTG*CG*GGAAG***CG***GG*CG*T*CG*TT*CG*GATTTAG  GATATTATAGTTTTTTTATGCGATTTTTAGAGTTCGTTTTATTT***CG***GG  GAATGTAGGT*CG*GGGTAGTAGGGAAGTTTTTAGGGATGTAGGGAG | 334bp | -4214～-3879 |
| Tapasin | GAAAAGTAAGGTTAGGTGTGGTGGTTTA***CG***TTTGTAATTTTAGAATTT  TGGGATAT***CG***AGGT***CG***GTGGATTATTTGAGGTTAGGAGTTTGAGATTA  GTTTGGTTAATA***CG***GTGAAATTT***CG***TTTTTATTAATATTAAAATTAG  TCGGGTATGGTGGCGGGTGTTTGTAATTTTAGTTA***CG***AGGGAGGTTGA  GGTAGGAGAAT***CG***TTTGAATT***CG***GAAGGTAGAGGTTG***CG***GTGAGT***CG***  AGATTATATTATGGTATTTTAGTTTGGGTAATAAG | 273bp | +3285～+3012 |
| ERp57 | AAGATTTAGGGTTTTTTGAAATAAAAGGGGTTAAGAGTGGTAAAGAT  ATTGAGTAGT***CG***ATTAAGTGGTTAGGTATTTTTATTTTGTGA***CG***GTA  TTATTAATTAAAAGTTTATTAAAAAAAAAAAAAAAAAAAAAAAAAGT  T***CG***GG***CG***TGGTGGTTTA***CG***TTTGTAATTTTAGTATTTTGGAATGT***CG***  AGG***CG***AG***CG***GATTA***CG***AGGTTAGGAGATAGAGATTATTTTGGTTAAT  A***CG***GTGAAATT***CG***GTTTTTATTAAAAATATAAAAAATTAGT***CG***GG***CG*** TGGTGG***CG***GG***CG***TTTGTAGGTTTAGTTATT***CG***GGAAGTTGAGGTAGGA  GAATGG***CG***TGAATT***CG***GGAGGTAGAGTTTGTAGTGA | 259bp | -1063～-804 |
